# Association between apolipoprotein C-III levels and coronary calcification detected by intravascular ultrasound in patients who underwent percutaneous coronary intervention

**DOI:** 10.3389/fcvm.2024.1430203

**Published:** 2024-08-21

**Authors:** Tatsuya Fukase, Tomotaka Dohi, Ryota Nishio, Mitsuhiro Takeuchi, Norihito Takahashi, Yuichi Chikata, Hirohisa Endo, Shinichiro Doi, Hiroki Nishiyama, Iwao Okai, Hiroshi Iwata, Seiji Koga, Shinya Okazaki, Katsumi Miyauchi, Hiroyuki Daida, Tohru Minamino

**Affiliations:** ^1^ Department of Cardiovascular Biology and Medicine, Juntendo University Graduate School of Medicine, Bunkyo-ku, Japan; ^2^ Department of Radiological Technology, Faculty of Health Science, Juntendo University Graduate School, Bunkyo-ku, Japan; ^3^ Japan Agency for Medical Research and Development-Core Research for Evolutionary Medical Science and Technology (AMED-CREST), Japan Agency for Medical Research and Development, Chiyoda-ku, Japan

**Keywords:** apolipoprotein C-III (ApoC-III), coronary calcification, calcified nodules, intravascular ultrasound (IVUS), percutaneous coronary intervention

## Abstract

There are few reports on the association between apolipoprotein C-III (ApoC-III) and coronary calcification using intravascular modalities. This study aimed to investigate the impacts of ApoC-III levels on coronary calcification using grayscale intravascular ultrasound (IVUS). Consecutive 263 culprit lesions for 202 patients who underwent percutaneous coronary intervention using grayscale IVUS were included in this study and divided into four groups based on quartile ApoC-III values. This study assessed plaque characteristics, including severe calcification (>180° arc) at the minimum lumen area site and presence of calcified nodules within the culprit lesion using grayscale IVUS, and evaluated whether ApoC-III levels were associated with coronary calcified plaques. The highest ApoC-III quartile [Quartile 4 (Q4)] had a higher proportion of complex lesions, calcified plaques, severe calcification, calcified nodules, plaque burden, and total atheroma volume than the lowest ApoC-III quartile [Quartile 1 (Q1)]. Additionally, multivariable logistic regression analysis showed that Q4 was significantly associated with severe calcification and calcified nodules, with Q1 as the reference (odds ratio [OR]: 2.70, 95% confidence intervals [CIs]: 1.04–7.00, *p *= 0.042; and OR: 3.72, 95% CIs 1.26–11.0, *p *= 0.017, respectively). Furthermore, ApoC-III level (1-mg/dl increase) was a strong significant predictor of severe calcification (OR: 1.07, 95% CIs: 1.00–1.15, *p *= 0.040) and calcified nodules (OR: 1.09, 95% CIs: 1.01–1.19, *p *= 0.034) according to the multivariable logistic regression analysis. This study is the first to verify that elevated ApoC-III levels are associated with the development of severe calcification and progression to calcified nodules as detected by grayscale IVUS.

## 1 Introduction

Vascular calcification can occur in various arteries and affect the intimal and/or medial layer of the vessels. Intimal calcification is associated with coronary atherosclerotic lesions, and is caused by factors such as advanced age, sex differences, hypertension, diabetes mellitus, dyslipidemia, cigarette smoking, and kidney disease (1). Coronary artery calcification (CAC) is a characteristic feature of coronary atherosclerosis and can independently predict future cardiovascular events, providing additional information beyond traditional coronary risk factors (2, 3). CAC is influenced by lipoproteins, which are known to play a role in the development of atherosclerotic plaques, and especially low-density lipoprotein cholesterol (LDL-C) has been identified as a significant risk factor for CAC (4). In addition, high-density lipoprotein cholesterol and very low-density lipoprotein, which contains apolipoprotein C-III (ApoC-III), also contribute to CAC progression (5, 6). ApoC-III alone or as a component of very low-density lipoprotein activates and adheres monocytes to endothelial cells, playing a causal role in the development of atherosclerotic lesions (7, 8). Some studies have investigated the relationship between ApoC-III and CAC using non-invasive imaging examinations. These studies have found that elevated ApoC-III levels are associated with increased CAC, and ApoC-III deficiency resulting from genetic mutations is associated with reduced CAC (9, 10). However, there are no reports confirming these relationships using an intravascular modality that is capable of detecting intimal coronary calcification. Therefore, this study aimed to investigate the impact of ApoC-III levels on coronary calcification detected by grayscale intravascular ultrasound (IVUS) in patients who underwent percutaneous coronary intervention (PCI).

## 2 Material and methods

### 2.1 Study design and population

**Figure 1:**
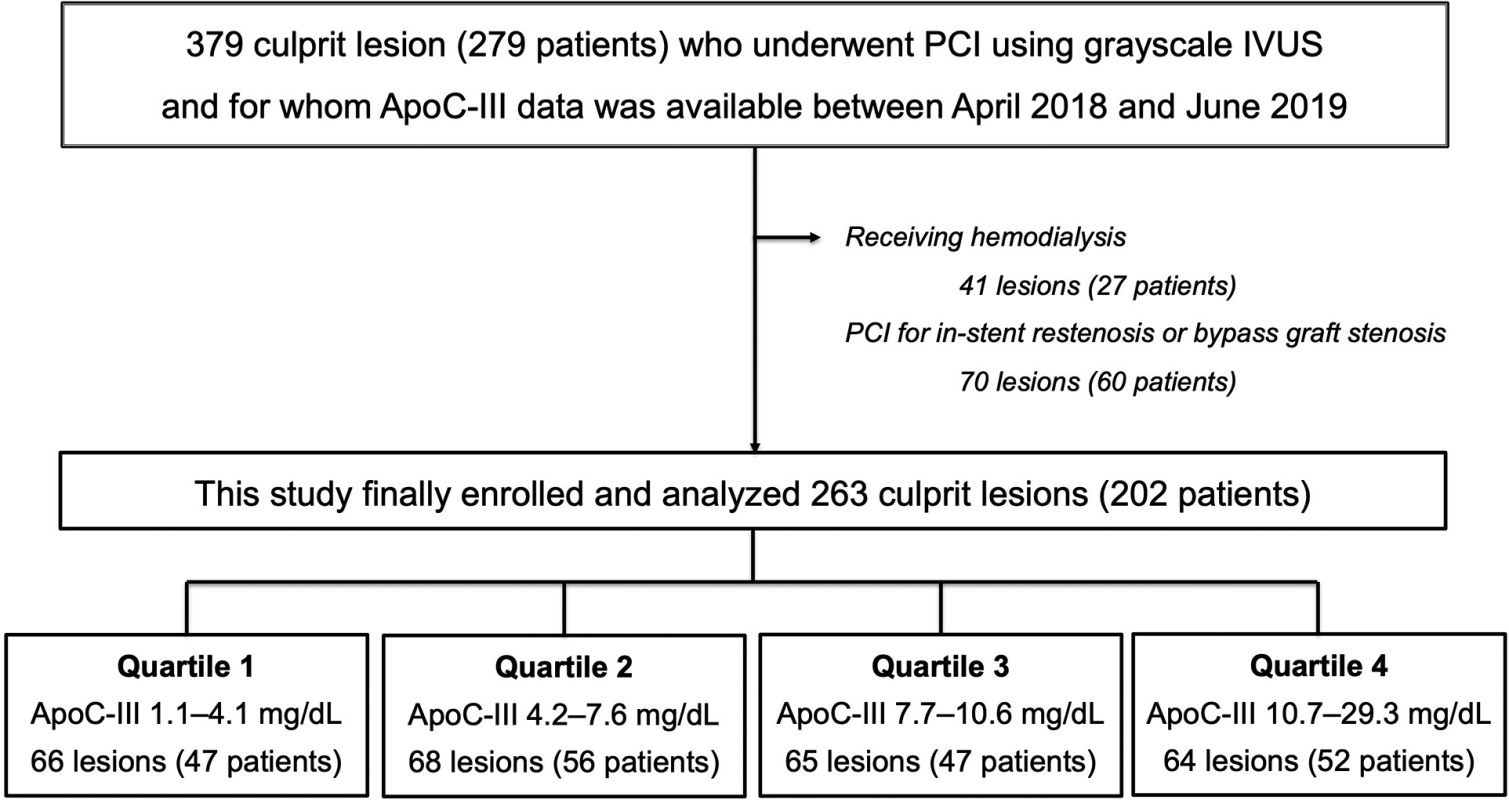
Flow chart. Among the 279 patients (379 culprit lesions) who underwent PCI using grayscale IVUS and for whom ApoC-III data were available between April 2018 and June 2019, 27 patients who received hemodialysis and 60 patients who underwent PCI for in-stent restenosis or bypass graft stenosis were excluded from this study. Finally data of 263 culprit lesions (202 patients) were divided into four groups based on the quartile value of ApoC-III and were analyzed. Overall, 66 lesions (47 patients) were allocated to Quartile 1 in the range of ApoC-III 1.1–4.1 mg/dl, 68 lesions (56 patients) were allocated to Quartile 2 in the range of ApoC-III 4.2–7.6 mg/dl, 65 lesions (47 patients) were allocated to Quartile 3 in the range of ApoC-III 7.7–10.6 mg/dl, and 64 lesions (52 patients) were allocated to Quartile 4 in the range of ApoC-III 10.7–29.3 mg/dl. ApoC-III, apolipoprotein C-III; IVUS, intravascular ultrasound; PCI, percutaneous coronary intervention.

In this single-center, cross-sectional study, we enrolled 379 culprit lesions in 279 consecutive patients who underwent PCI using grayscale IVUS and for whom ApoC-III data were available between April 2018 and June 2019. The following patients were excluded from this study: (1) 41 lesions in 27 patients receiving hemodialysis and (2) 70 lesions in 60 patients who underwent PCI for in-stent restenosis or bypass graft stenosis, as shown in Figure 1.

The ethics committee of the Juntendo Clinical Research and Trial Center approved this study (reference number: E22-0409), and all participants provided written informed consent. This study was conducted according to the principles of the Declaration of Helsinki (11).

### 2.2 Data collection and definitions

We collected data on patient's characteristics from an institutional database. Blood samples were collected in the morning, 1 day before the intervention, and after overnight fasting. All blood tests were performed at the same laboratory.

Patients with blood pressure levels >140/90 mmHg or those receiving antihypertensive drugs were considered hypertensive (12). Dyslipidemia was defined as a triglyceride level of ≥150 mg/dl, a low-density lipoprotein cholesterol level of ≥140 mg/dl, a high-density lipoprotein cholesterol level of <40 mg/dl, or the administration of lipid-lowering therapy (13). Diabetes mellitus was defined as a hemoglobin A1c level of ≥6.5%, the administration of oral hypoglycemic agents, or insulin injection (14). Anemia was defined based on the hemoglobin levels recommended by the World Health Organization (<13.0 g/dl in men and <12.0 g/dl in women) (15). A family history of premature coronary artery disease was defined as the presence of any first-degree relative with premature cardiovascular disease (age <55 years for men and <65 years for women) (16). Chronic kidney disease was defined as an estimated glomerular filtration rate of <60 ml/min/1.73 m^2^ based on the Modification of Diet in Renal Disease equation, modified using the baseline serum creatinine level (17). Coronary artery lesion types (A, B1, B2, and C) were defined according to the American Heart Association/American College of Cardiology classification (18).

Regarding lipid profiles, total cholesterol, high-density lipoprotein cholesterol, LDL-C, and triglyceride levels were assayed using LABOSPECT 008*α* (Hitachi, Ltd., Tokyo, Japan). Lipoprotein(a) levels were measured using a latex agglutination–turbidimetric immunoassay, and apolipoprotein levels were simultaneously measured using a turbidimetric immunoassay. We used the Apo Auto・N “Daiichi” measurement kit (Sekisui Medical Co., Ltd., Tokyo, Japan) to assess apolipoprotein levels. The reference standard range for ApoC-III was 5.5–9.5 mg/dl, with an absorbance of 0.20–0.55 per 7.5 mg/dl of ApoC-III. The accuracy of sensitivity was 80%–120% of the expected assay value, and the within-run reproducibility had a coefficient of variation ≤5% (19).

### 2.3 IVUS imaging acquisition and analysis

An IVUS catheter was inserted distal to the culprit lesion and pulled back at a rate of 0.5 mm/s, after intracoronary nitroglycerin injection. IVUS imaging of the culprit lesion segment was performed before balloon dilatation or after small balloon dilatation (1.5–2.0 mm). Pre- and post-PCI IVUS findings were compared to determine the stenting location for the culprit lesion segment. Grayscale IVUS was performed using two commercially available systems: OptiCross™ (Boston Scientific, Marlborough, MA, USA) and AltaView™ (Terumo, Tokyo, Japan). The analysis of the IVUS images was performed by two cardiologists (T.F. and T.D.). Various quantitative grayscale IVUS measurements were obtained, including lumen cross-sectional area (CSA), external elastic membrane CSA, plaque and media CSA, plaque burden, remodeling index, plaque characteristics, plaque rupture, thrombus, ultrasound attenuation, and maximum angle of the ultrasound attenuation. Atheroma classification included fibrous plaques characterized by intermediate echogenicity between soft (hypoechoic) plaques and highly echogenic calcified plaques, and calcified plaques displayed as higher echogenicity than the adventitia with an acoustic shadow. Quantitative grayscale IVUS measurements were performed using QIvus version 2.1 (Medis, Leiden, Netherlands), following a clinical expert consensus document (20). This software can also measure vessel volume, total atheroma volume, and percentage atheroma volume.

### 2.4 Endpoints

This study focused on coronary calcification detected by grayscale IVUS. The endpoints were (1) severe calcification at the minimum lumen area (MLA) site, defined as a calcification angle of >180° (21); (2) the presence of calcified nodules within the culprit lesion, which are identified as distinct calcification with an irregular, protruding and convex liminal surface (20).

### 2.5 Statistical analysis

All data were analyzed using JMP® Pro, version 16.0.0 for Macintosh (SAS Institute, Cary, NC, USA). Probabilities were expressed as two-tailed values, with statistical significance set at *p *< 0.05. Confidence intervals (CIs) were computed at the 95% level.

Categorical data were presented as numbers (percentage) and were compared using the Chi-square test. Continuous variables were expressed as mean ± standard deviation or median (interquartile range) and compared using a one-way analysis of variance or the Kruskal–Wallis test. The Shapiro–Wilk test was used to examine whether the scores were likely to follow a certain distribution in all patients. If *p *< 0.05, the variables were considered not normally distributed.

Multivariable logistic regression analysis was performed to predict severe calcification at the MLA site, and calcified nodules within the culprit lesion were analyzed to compare the effects of each group as a reference for the lowest ApoC-III quartile group. The analysis was adjusted for the following covariates: age, sex, body mass index, hypertension, LDL-C levels, diabetes mellitus, smoking, family history of premature coronary artery disease, chronic kidney disease, anemia, high-sensitivity C-reactive protein, acute coronary syndrome, multivessel disease and total atheroma volume, which have a causal relationship with CAC (1, 22, 23). Similarly, multivariable logistic regression analysis was performed using a stepwise selection of these covariates with entry/stay criteria of 0.20/0.20 to evaluate the impact of ApoC-III as a continuous variable.

There are no previous studies that have verified the relationship between ApoC-III levels and calcified plaques detected by IVUS; thus, this study retrospectively analyzed the sample size using a post-hoc analysis. As a result, the post-hoc power analysis for the impacts of ApoC-III levels on severe calcification (*α *= 0.05, standard deviation = 4.34, effect size = 0.494) and progression to calcified nodules (*α *= 0.05, standard deviation = 4.34, effect size = 0.425) showed that the minimum sample size required was 298 and 403, respectively.

## 3 Results

### 3.1 Patient's clinical characteristics

In total, 263 culprit lesions in 202 patients who underwent PCI using grayscale IVUS were analyzed. The participants were divided into four groups based on the quartile value of ApoC-III, as shown in Figure 1. The distribution of ApoC-III levels with a median value of 7.6 mg/dl is presented in Supplementary Figure S1.

Table 1 summarizes the baseline clinical characteristics of the patients. The mean age of the patients was 69 ± 11 years, and 78% were men. Half of the patients had diabetes mellitus, with 79 patients taking oral hypoglycemic agents and 20 patients receiving insulin injections. Approximately 70% of patients had a smoking history, and the prevalence of chronic kidney disease and anemia was 36% and 31%, respectively. In total, 176 patients (87%) were taking statins, with a mean LDL-C value of 83 mg/dl. The highest ApoC-III quartile group [Quartile 4 (Q4)] had significantly higher body mass index and total cholesterol, LDL-C, triglycerides, apolipoprotein B, apolipoprotein E, and fasting blood glucose levels, as well as a younger age and a lower prevalence of hypertension than the lowest ApoC-III quartile group [Quartile 1 (Q1)].

**Table 1:** Patients’ baseline clinical characteristics.

	Overall *n* = 202	Quartile 1 (1.1–4.1 mg/dl) *n* = 47	Quartile 2 (4.2–7.6 mg/dl) *n* = 56	Quartile 3 (7.7–10.6 mg/dl) *n* = 47	Quartile 4 (10.7–29.3 mg/dl) *n* = 52	*p* value
Clinical characteristics						
Age, years	69 ± 11	71 ± 12	69 ± 11	70 ± 11	66 ± 11	0.150
Male, *n* (%)	157 (78)	34 (72)	45 (80)	36 (77)	42 (81)	0.729
BMI, kg/m^2^	24.6 ± 3.1	23.5 ± 3.6	24.4 ± 2.7	24.8 ± 2.8	25.5 ± 3.2	0.012
Systolic BP, mmHg	125 ± 14	126 ± 13	121 ± 12	127 ± 13	124 ± 16	0.157
Diastolic BP, mmHg	68 ± 8	68 ± 8	66 ± 8	69 ± 9	68 ± 9	0.502
TC level, mg/dl	155 ± 29	146 ± 36	148 ± 25	155 ± 28	170 ± 27	<0.001
LDL-C level, mg/dl	83 ± 24	78 ± 28	80 ± 19	83 ± 24	91 ± 25	0.048
Triglyceride level, mg/dl	132 ± 61	99 ± 40	111 ± 50	122 ± 40	193 ± 81	<0.001
HDL-C level, mg/dl	47 ± 13	48 ± 13	46 ± 12	48 ± 14	46 ± 15	0.782
Lipoprotein (a) level, mg/dl	17 (9, 38)	23 (12, 40)	13 (6, 36)	17 (10, 37)	16 (9, 43)	0.291
ApoA-I level, mg/dl	123 ± 25	117 ± 29	123 ± 26	127 ± 21	127 ± 23	0.164
ApoB level, mg/dl	77 ± 17	71 ± 17	72 ± 15	85 ± 17	91 ± 17	<0.001
ApoE level, mg/dl	3.5 (3.0, 4.3)	3.1 (2.6, 3.5)	3.4 (2.9, 4.0)	3.8 (3.1, 4.4)	4.4 (3.7, 5.6)	<0.001
ApoC-III level, mg/dl	7.6 (4.2, 10.7)	3.0 (2.3, 3.6)	6.2 (5.2, 7.0)	9.0 (8.2, 9.8)	13.0 (11.2, 15.6)	<0.001
Estimated GFR, ml/min/L.73 m^2^	66.1 ± 19.1	67.6 ± 18.9	66.4 ± 19.7	68.0 ± 18.8	62.7 ± 18.9	0.496
FBG level, mg/dl	99 (89, 120)	95 (85, 112)	102 (88, 120)	98 (88, 125)	100 (95, 128)	0.093
HbA1c level, %	6.2 (5.8, 7.0)	6.1 (5.7, 6.8)	6.2 (5.8, 6.9)	6.1 (5.8, 7.1)	6.3 (6.0, 7.3)	0.234
Hs-CRP level, mg/dl	0.07 (0.03–0.18)	0.08 (0.04–0.22)	0.05 (0.02–0.13)	0.07 (0.03–0.25)	0.07 (0.04–0.17)	0.100
LVEF, %	67 (60, 70)	67 (55, 70)	66 (58, 70)	68 (62, 70)	68 (59, 71)	0.693
Comorbidity						
Hypertension, *n* (%)	184 (91)	46 (98)	49 (88)	44 (94)	45 (87)	0.106
Dyslipidemia, *n* (%)	196 (97)	44 (94)	54 (96)	46 (98)	52 (100)	0.190
Diabetes mellitus, *n* (%)	101 (50)	20 (43)	30 (54)	34 (51)	27 (52)	0.698
Smoking history, *n* (%)	137 (68)	34 (72)	39 (70)	26 (55)	38 (73)	0.221
Family history of premature CAD, *n* (%)	48 (24)	9 (19)	18 (33)	11 (23)	10 (20)	0.322
Chronic kidney disease, *n* (%)	72 (36)	14 (30)	23 (41)	13 (28)	22 (42)	0.288
Anemia, *n* (%)	82 (31)	15 (32)	17 (30)	16 (34)	13 (25)	0.782
Acute coronary syndrome, *n* (%)	17 (6)	3 (5)	6 (9)	5 (8)	3 (5)	0.675
Medication						
β-blocker, *n* (%)	117 (58)	32 (68)	35 (63)	20 (43)	30 (58)	0.071
Calcium channel blocker, *n* (%)	101 (50)	21 (45)	25 (45)	27 (57)	28 (54)	0.470
RAS-i, *n* (%)	115 (57)	23 (49)	38 (68)	26 (55)	28 (54)	0.234
Statin, *n* (%)	176 (87)	38 (81)	49 (88)	43 (91)	46 (88)	0.485
Aspirin, *n* (%)	192 (95)	46 (98)	52 (93)	46 (98)	48 (92)	0.358
Oral hypoglycemic agents, *n* (%)	79 (39)	14 (30)	24 (43)	18 (38)	23 (44)	0.443
Insulin, *n* (%)	20 (10)	2 (4)	9 (16)	2 (4)	7 (13)	0.075

ApoA-I, apolipoprotein A-I; ApoB, apolipoprotein B; ApoC-III, apolipoprotein C-III; ApoE, apolipoprotein E; BMI, body mass index; BP, blood pressure; CAD, coronary artery disease; FBG, fasting blood glucose; GFR, glomerular filtration rate; HbA1c, hemoglobin A1c; HDL-C, high-density lipoprotein cholesterol; Hs-CRP, high-sensitivity C-reactive protein; LDL-C, low-density lipoprotein cholesterol; LVEF, left ventricular ejection fraction; RAS, renin-angiotensin system inhibitor; TC, total cholesterol.

### 3.2 Procedural and imaging characteristics

Table 2 presents the imaging characteristics of the lesions. In terms of angiographic and procedural characteristics, Q4 had a significantly higher proportion of complex lesions and longer lesion length compared to Q1. However, there were no significant differences between the two groups in terms of lesion site, lesion diameter, proportion of stents usage, and stent diameter and length. Q4 also had a significantly higher plaque burden, total atheroma volume, and percentage atheroma volume than Q1; however, the two groups did not differ in terms of MLA or remodeling index.

**Table 2:** Imaging characteristics of lesions.

	Overall *N* = 263	Quartile 1 (1.1–4.1 mg/dl) *N* = 66	Quartile 2 (4.2–7.6 mg/dl) *N* = 68	Quartile 3 (7.7–10.6 mg/dl) *N* = 65	Quartile 4 (10.7–29.3 mg/dl) *N* = 64	*p* value
Angiographic characteristics						
Lesion site						0.949
Right coronary artery, *n* (%)	64 (24)	13 (20)	15 (22)	19 (29)	17 (27)	
Left main coronary trunk, *n* (%)	5 (2)	1 (2)	2 (3)	1 (2)	1 (2)	
Left anterior descending artery, *n* (%)	138 (53)	36 (55)	37 (54)	30 (46)	35 (55)	
Left circumflex artery, *n* (%)	55 (21)	15 (23)	14 (21)	15 (23)	11 (17)	
Lesion classification						0.061
Type A, *n* (%)	7 (3)	1 (2)	3 (4)	0 (0)	3 (5)	
Type B1, *n* (%)	33 (13)	15 (23)	8 (12)	7 (11)	3 (5)	
Type B2, *n* (%)	92 (35)	21 (32)	21 (31)	27 (42)	23 (36)	
Type C, *n* (%)	131 (50)	29 (44)	31 (53)	31 (48)	35 (55)	
Reference diameter, mm	2.8 ± 0.5	2.9 ± 0.6	2.7 ± 0.5	2.8 ± 0.5	2.8 ± 0.5	0.559
Lesion length, mm	18.0 (13.0, 28.0)	18.5 (10.0, 24.0)	18.0 (13.0, 32.0)	18.0 (15.0, 26.0)	18.5 (13.0, 31.8)	0.702
IVUS findings						
Pull-back length, mm	49.8 (36.8, 63.6)	42.8 (35.0, 61.1)	51.1 (38.3, 62.1)	53.7 (38.6, 69.4)	50.3 (38.5, 69.1)	0.227
Lesion length, mm	28.7 (20.2, 42.8)	26.4 (16.4, 36.5)	33.9 (19.3, 47.6)	27.4 (21.6, 37.2)	30.6 (20.5, 47.1)	0.076
Plaque morphology						
Fibrous plaque, *n* (%)	113 (43)	31 (47)	34 (50)	26 (36)	22 (34)	0.262
Soft plaque, *n* (%)	68 (26)	22 (33)	15 (22)	15 (23)	16 (25)	0.450
Calcified plaque, *n* (%)	162 (62)	32 (48)	38 (56)	44 (68)	48 (75)	0.008
Severe calcification at MLA site, *n* (%)	60 (23)	9 (14)	13 (19)	19 (29)	19 (30)	0.068
Max arc of calcification at MLA site, °	140 (99, 230)	143 (93, 216)	136 (102, 286)	155 (104, 277)	136 (96, 231)	0.896
Calcified nodule, *n* (%)	50 (19)	7 (11)	12 (18)	15 (23)	16 (25)	0.132
Plaque rupture, *n* (%)	56 (21)	15 (23)	14 (21)	11 (17)	16 (25)	0.709
Thrombus, *n* (%)	41 (16)	10 (15)	14 (21)	6 (9)	11 (17)	0.307
Ultrasound attenuation, *n* (%)	114 (43)	32 (48)	24 (35)	26 (40)	32 (50)	0.264
Max arc of attenuation, °	126 (85, 174)	124 (88, 157)	106 (80, 175)	107 (86, 149)	158 (92, 187)	0.303
MLA, mm^2^	1.8 (1.6, 2.2)	1.9 (1.6, 2.5)	1.7 (1.5, 2.0)	1.9 (1.6, 2.4)	1.8 (1.5, 2.1)	0.057
EEM CSA at MLA site, mm^2^	10.4 (7.4, 14.9)	10.4 (7.1, 13.0)	9.9 (7.4, 15.0)	11.0 (8.4, 15.0)	10.8 (7.1, 16.4)	0.497
Plaque burden at MLA site, %	80.2 ± 9.4	77.9 ± 10.5	80.7 ± 9.7	80.7 ± 8.9	81.5 ± 8.3	0.137
Remodeling index at MLA site	0.98 ± 0.06	0.98 ± 0.05	0.99 ± 0.05	0.98 ± 0.07	0.99 ± 0.05	0.801
Vessel volume, mm^3^	350.5 (236.0, 501.1)	288.6 (221.0, 422.5)	384.4 (257.8, 501.2)	343.2 (216.1, 474.4)	400 (282.1, 611.1)	0.037
Total atheroma volume, mm^3^	228.0 (141.1, 330.1)	183.9 (118.2, 267.4)	245.7 (157.8, 343.9)	218.5 (132.7, 323.6)	260.5 (151.8, 405.3)	0.014
Percent atheroma volume, %	63.8 ± 7.9	60.8 ± 6.9	64.6 ± 8.9	65.1 ± 7.2	64.8 ± 8.2	0.004
Procedural characteristics						
Use of drug-eluting stent, *n* (%)	251 (97)	64 (98)	64 (94)	62 (97)	61 (97)	0.587
Stent diameter, mm	2.8 ± 0.4	2.8 ± 0.4	2.8 ± 0.4	2.8 ± 0.5	2.8 ± 0.4	0.639
Stent length, mm	33 (23, 48)	32 (20, 38)	38 (23, 55)	30 (23, 38)	35 (26, 53)	0.056

CSA, cross sectional area; EEM, external elastic membrane; IVUS, intravascular ultrasound; MLA, minimum lumen area.

In the overall qualitative analysis, fibrous, soft, and calcified plaques at the MLA site accounted for 43%, 26%, and 62%, respectively. Q3 and Q4 had a significantly higher frequency of calcified plaques compared to Q1 (68% vs. 48%, *p *= 0.025% and 75% vs. 48%, *p *= 0.002), but there were no significant differences in fibrous and soft plaques. Additionally, there were no significant differences between the groups in terms of plaque rupture, thrombus, and ultrasound attenuation. In contrast, severe calcification at the MLA site was more frequently observed in Q3 and Q4 than in Q1 (29% vs. 14%, *p *= 0.028% and 30% vs. 14%, *p *= 0.025). Furthermore, Q4 tended to have a higher frequency of calcified nodules within the culprit lesion compared to Q1 (25% vs. 11%, *p *= 0.030). A representative case of an IVUS-detected coronary calcification is shown in Supplementary Figure S2.

### 3.3 Impacts of an elevated ApoC-III level on coronary calcification

Table 3 presents the results of multivariable logistic regression analysis adjusted for various covariates that have a causal relationship with CAC, with Q1 as the reference. The analysis revealed a significant association between Q4 and severe calcification when compared to Q1 [odds ratio (OR): 2.69, 95% CIs: 1.01–7.13, *p *= 0.047]. Similarly, Q4 was significantly associated with calcified nodules compared to Q1 (OR: 3.28, 95% Cls: 1.08–10.0, *p *= 0.037). In addition, Table 4 shows the results of multivariable logistic regression analysis, which revealed that ApoC-III level (1 mg/dl increase) (OR: 1.07, 95% CIs: 1.00–1.15, *p *= 0.040), age (1-year increase) (OR: 1.03, 95% CIs: 1.00–1.06, *p *= 0.039), and total atheroma volume (10 mm^3^ increase) (OR: 1.01, 95% CIs: 1.00–1.02, *p *= 0.042) were strong independent predictors of severe calcification at the MLA site. Similarly, Table 4 shows the results of multivariable logistic regression analysis, which revealed that ApoC-III level (1 mg/dl increase) (OR: 1.09, 95% CIs: 1.01–1.19, *p *= 0.034), age (1-year increase) (OR: 1.06, 95% CIs: 1.02–1.10, *p *= 0.004), total atheroma volume (10 mm^3^ increase) (OR: 1.03, 95% CIs: 1.01–1.05, *p *= 0.002), and anemia (OR: 2.16, 95% CIs: 1.00–4.67, *p *= 0.049) were strong independent predictors of calcified nodules within the culprit lesion.

**Table 3:** Impacts of elevated apoC-III levels on severe calcification at MLA site and calcified nodules within culprit lesion.

Covariates	Severe calcification at MLA site		Calcified nodules within culprit lesion	
	Odds ratio (95% confidence intervals)	*p* value	Odds ratio (95% confidence intervals)	*p* value
	Unadjusted model		Unadjusted model	
Quartile 1 (1.1–4.1 mg/dl)	Reference	–	Reference	–
Quartile 2 (4.2–7.6 mg/dl)	1.50 (0.59–3.78)	0.394	1.81 (0.66–4.92)	0.247
Quartile 3 (7.7–10.6 mg/dl)	2.62 (1.08–6.33)	0.033	2.53 (0.96–6.69)	0.062
Quartile 4 (10.7–29.3 mg/dl)	2.67 (1.10–6.47)	0.029	2.81 (1.07–7.39)	0.036
	Adjusted model		Adjusted model	
Quartile 1 (1.1–4.1 mg/dl)	Reference	–	Reference	–
Quartile 2 (4.2–7.6 mg/dl)	1.46 (0.54–3.98)	0.460	1.66 (0.53–5.23)	0.383
Quartile 3 (7.7–10.6 mg/dl)	2.20 (0.85–5.67)	0.102	2.58 (0.85–7.78)	0.093
Quartile 4 (10.7–29.3 mg/dl)	2.69 (1.01–7.13)	0.047	3.28 (1.08–10.0)	0.037

Adjusted for age, sex, body mass index, hypertension, LDL-C levels, diabetes mellitus, smoking, family history of premature CAD, chronic kidney disease, anemia, high-sensitivity C-reactive protein, acute coronary syndrome, multivessel disease and total atheroma volume.

ApoC-III, apolipoprotein C-III; CAD, coronary artery disease; LDL-C, low-density lipoprotein cholesterol; MLA, minimum lumen area.

**Table 4:** Univariable and multivariable logistic regression analysis for prediction of severe calcification and calcified nodules.

	Severe calcification at MLA site				Calcified nodules within culprit lesion			
	Univariable		Multivariable		Univariable		Multivariable	
	OR (95% CIs)	*p* value	OR (95% CIs)	*p* value	OR (95% CIs)	*p* value	OR (95% CIs)	*p* value
Age, 1-year increase	1.02 (0.99–1.05)	0.096	1.03 (1.00–1.06)	0.039	1.04 (1.01–1.07)	0.021	1.06 (1.02–1.10)	0.004
ApoC-III, 1 mg/dl increase	1.06 (0.99–1.13)	0.069	1.07 (1.00–1.15)	0.040	1.06 (0.99–1.13)	0.116	1.09 (1.01–1.19)	0.034
TAV, 10 mm^3^ increase	1.01 (1.00–1.03)	0.039	1.01 (1.00–1.03)	0.042	1.02 (1.01–1.04)	0.003	1.03 (1.01–1.05)	0.002
Acute coronary syndrome	1.94 (0.69–5.48)	0.212	2.21 (0.76–6.42)	0.145	0.25 (0.03–1.94)	0.185		
Anemia	2.65 (1.46–4.80)	0.001			2.00 (1.06–3.77)	0.032	2.16 (1.00–4.67)	0.049
Multivessel disease	1.12 (0.56–2.24)	0.750			2.09 (0.89–4.91)	0.092	2.55 (0.99–6.59)	0.053
Family history of premature CAD	0.93 (0.47–1.83)	0.823			1.48 (0.74–2.94)	0.264	1.99 (0.91–4.35)	0.086
Hs-CRP, 0.1 mg/dl increase	1.00 (0.96–1.05)	0.907			0.96 (0.90–1.03)	0.248	0.94 (0.87–1.01)	0.116
Smoking	0.72 (0.40–1.32)	0.291			1.17 (0.60–2.28)	0.651	1.86 (0.85–4.06)	0.120
Hypertension	0.88 (0.31–2.52)	0.809			0.68 (0.24–1.97)	0.480	0.42 (0.13–1.41)	0.161
Chronic kidney disease	1.49 (0.83–2.67)	0.187			1.10 (0.58–2.09)	0.759	0.58 (0.26–1.28)	0.175
Diabetes mellites	1.47 (0.82–2.63)	0.194			1.28 (0.69–2.39)	0.428		
LDL-C, 10 mg/dl increase	0.98 (0.86–1.10)	0.700			1.02 (0.89–1.16)	0.818		
BMI, 1 kg/m^2^ increase	0.99 (0.90–1.09)	0.832			0.97 (0.88–1.08)	0.584		
Male	0.97 (0.48–1.96)	0.936			1.53 (0.67–3.47)	0.312		

ApoC-III, apolipoprotein C-III; BMI, body mass index; CAD, coronary artery disease; CIs, confidence intervals; Hs-CRP, high-sensitivity C-reactive protein; LDL-C, low-density lipoprotein cholesterol; MLA, minimum lumen area; OR, odds ratio; TAV, total atheroma volume.

## 4 Discussion

To the best of our knowledge, this study is the first to verify the association between ApoC-III levels and coronary calcification using grayscale IVUS. The highest quartile ApoC-III group showed a higher frequency of calcified plaques and severe calcification at the MLA site, as well as the presence of calcified nodules within the culprit lesion. Additionally, this group exhibited higher plaque burden, total atheroma volume, and percentage atheroma volume compared to the lowest quartile ApoC-III group. In addition, when compared to the lowest quartile ApoC-III group, multivariable logistic regression analysis revealed a significant association between the highest quartile ApoC-III group and the development of severe calcification and progression to calcified nodules. Furthermore, an elevated ApoC-III level emerged a strong independent predictor of severe calcification at the MLA site and the presence of calcified nodules within the culprit lesion. Currently, as “the lower, the better” is proposed, aggressive LDL-C lowering therapy represented by statins is recommended as secondary prevention for cardiovascular disease (24). A meta-analysis found that statins promote plaque calcification with a stabilizing effect (25). Other analyses have shown that statins can reduce vulnerable plaque volume while increasing calcification and fibrous cap development (26, 27). Statins may increase coronary calcification within the necrotic core to stabilize unstable plaques and reduce future cardiovascular events. However, severe calcification is associated with poor outcomes. Some studies have found that heavily calcified lesions are significantly associated with target lesion failure, stent thrombosis, and all-cause mortality (28, 29). Calcified nodules, which are characterized by progressing from sheet to nodule, are also unstable. These nodules have more necrotic core and less collagen calcification and degree of circumferential calcification (30, 31). Calcified nodules account for 2%–7% of acute coronary syndrome cases, and their progression is linked to worsening clinical outcomes, including cardiac death, acute coronary syndrome recurrence, and target lesion revascularization (32, 33). Notably, the presence and extent of CAC, assessed using computed tomography (CT), are known to be concurrent with the development of advanced atherosclerosis and have been established as predictors of future cardiovascular events (34–36). However, the CAC score measured by CT alone may not provide sufficient information to understand the degree of complexity and differences in the location of coronary calcification. In contrast, intravascular imaging modalities, such as grayscale IVUS, can provide more detailed information about intimal coronary calcification. Therefore, this study aimed to analyze the characteristics of coronary calcification using grayscale IVUS and investigate its relationship with ApoC-III, a biomarker associated with CT-derived CAC as reported in previous studies.

ApoC-III is primarily synthesized by the liver and to a lesser extent by enterocytes, where the human ApoC-III gene is expressed. The mature ApoC-III protein is made up of 79 amino acids and has a molecular mass of 8.8 kDa (37). Recent research has demonstrated that ApoC-III has a multifaceted impact on various pathophysiological processes, including triglyceride-rich lipoprotein metabolism, inflammatory responses, atherosclerosis progression, glucose metabolism, and cardiovascular diseases (38). As previously mentioned, ApoC-III plays a direct role in the development of atherosclerosis, and its levels are positively correlated with inflammatory cytokines such as tumor necrosis factor-α and interleukin-1β (39, 40). These inflammatory cytokines are responsible for inducing the expression of adhesion molecules such as vascular cell adhesion molecule-1, which leads to atherosclerosis and subsequently promotes calcification progression (41, 42). Additionally, ApoC-III increases the expression of vascular cell adhesion molecule-1 expression in human coronary artery endothelial cells, whereas statins can reduce ApoC-III-induced monocyte adhesion to endothelial cells (43). In the current study, lipid control was relatively successful, with a mean LDL-C level of 83 mg/dl. Therefore, it is possible that ApoC-III, as a residual risk factor, could impact the formation of atherosclerotic coronary calcification, including the development of severe calcification and the progression to calcified nodules.

In terms of pharmacological interventions for reducing ApoC-III levels, there have been reports on therapeutic agents that target triglycerides, such as fibrates, niacin, and omega-3 carboxylic acids. These agents have shown to reduce ApoC-III gene expression and ApoC-III levels by 10%–40% (44–46). Additionally, some studies have shown that volanesorsen, which inhibits the translation of ApoC-III messenger ribonucleic acid, can decrease ApoC-III levels by up to 70% in healthy individuals and 80% in patients with hyperglycemia (47, 48). Therefore, considering that LDL-C lowering therapy mainly represented by statins can effectively prevent atherosclerosis, these agents targeting ApoC-III may help suppress the development of severe calcification and the progression to calcified nodules.

### 4.1 Limitations

This study had some limitations that need to be considered. First, there may be unknown confounding factors that could have influenced the study outcomes, even with analytical adjustments. Second, the study only utilized grayscale IVUS to detect coronary calcified plaques. While IVUS can easily detect calcified plaques, it is unable to measure calcium thickness and volume. Furthermore, IVUS is highly sensitive and specific in detecting large dense calcified plaques or spotty calcifications, but its axial resolution (range 150–200 μm) is insufficient to visualize microcalcifications or thin-cap fibroatheroma, which are typically smaller than 65 μm (49). Third, IVUS is not able to distinguish the classification of calcified plaques, such as eruptive calcified nodules, superficial calcific sheets, and calcified protrusions, which can be identified by optical coherence tomography. Additionally, the presence of organized thrombus, which is difficult to identify even with optical coherence tomography, should also be considered as a limitation. Fourth, this study was limited to patients with coronary artery disease who underwent PCI for culprit lesions. Therefore, it remains unclear whether this causal relationship holds true for healthy subjects, patients eligible for primary prevention, and non-culprit lesions. Fifth, the results suggesting that ApoC-III levels could be a significant predictor of the residual risk of coronary calcification, which accelerates atherosclerosis, carry high clinical significance. However, the implications of CAC after LDL-C lowering therapy are currently being debated. Thus, in this study, it was not possible to accurately predict how ApoC-III levels might modulate the progression of atherosclerotic calcification as a surrogate marker in this study (25). Finally, although this study confirmed the impacts of ApoC-III levels on severe calcification and calcified nodules, the post-hoc power analysis for the impacts of ApoC-III levels on severe calcification and progression to calcified nodules showed that the minimum sample size required was 298 and 403, respectively. The sample size analyzed in this study was small; thus, further studies are needed to confirm the independent effects of ApoC-III on coronary calcification and establish a causal relationship between ApoC-III and coronary calcification.

## 5 Conclusions

This study is the first to report the association between ApoC-III levels and coronary calcification detected by grayscale IVUS. Elevated ApoC-III levels may be involved in the development of severe calcification and progression to calcified nodules.

## Data Availability

The raw data supporting the conclusions of this article will be made available by the authors, without undue reservation.

## Supplementary material

Supplementary Figure S1 Distribution of ApoC-III levels. Among the 202 patients, the ApoC-III showed a non-normal distribution with a median ApoC-III value of 7.6 mg/dl. ApoC-III, apolipoprotein C-III.

Supplementary Figure S2 A representative case. A 67-years-old male patient with chronic coronary syndrome showed severe stenosis of the left anterior descending artery segment 7 on angiography (middle panel). The IVUS images are shown in (**A**–**D**), and the presence of calcified nodules [**shown in (**A**,**B**)] and consecutive severe calcification at MLA (maximum calcification angle = 299°) were confirmed. IVUS, intravascular ultrasound; MLA, minimum lumen area.
