# Supplementary figures and images for: Association between apolipoprotein C-III levels and coronary calcification detected by intravascular ultrasound in patients who underwent percutaneous coronary intervention
Source: Front Cardiovasc Med. 2024 Aug 21;11:1430203. doi: 10.3389/fcvm.2024.1430203 (PMC11371589; doi:10.3389/fcvm.2024.1430203)

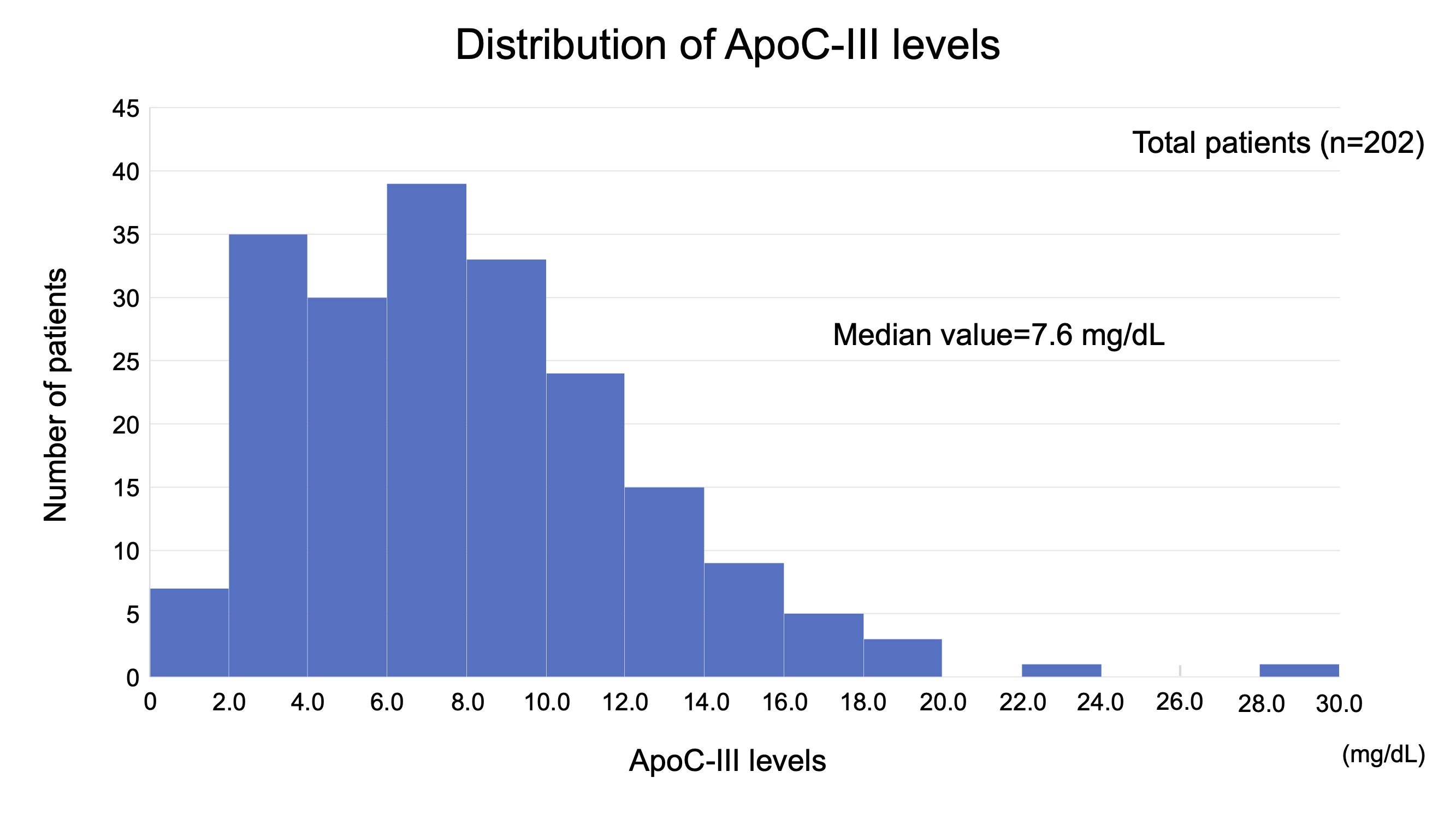

Supplement: Supplementary Figure S1 [file Image1.jpeg]

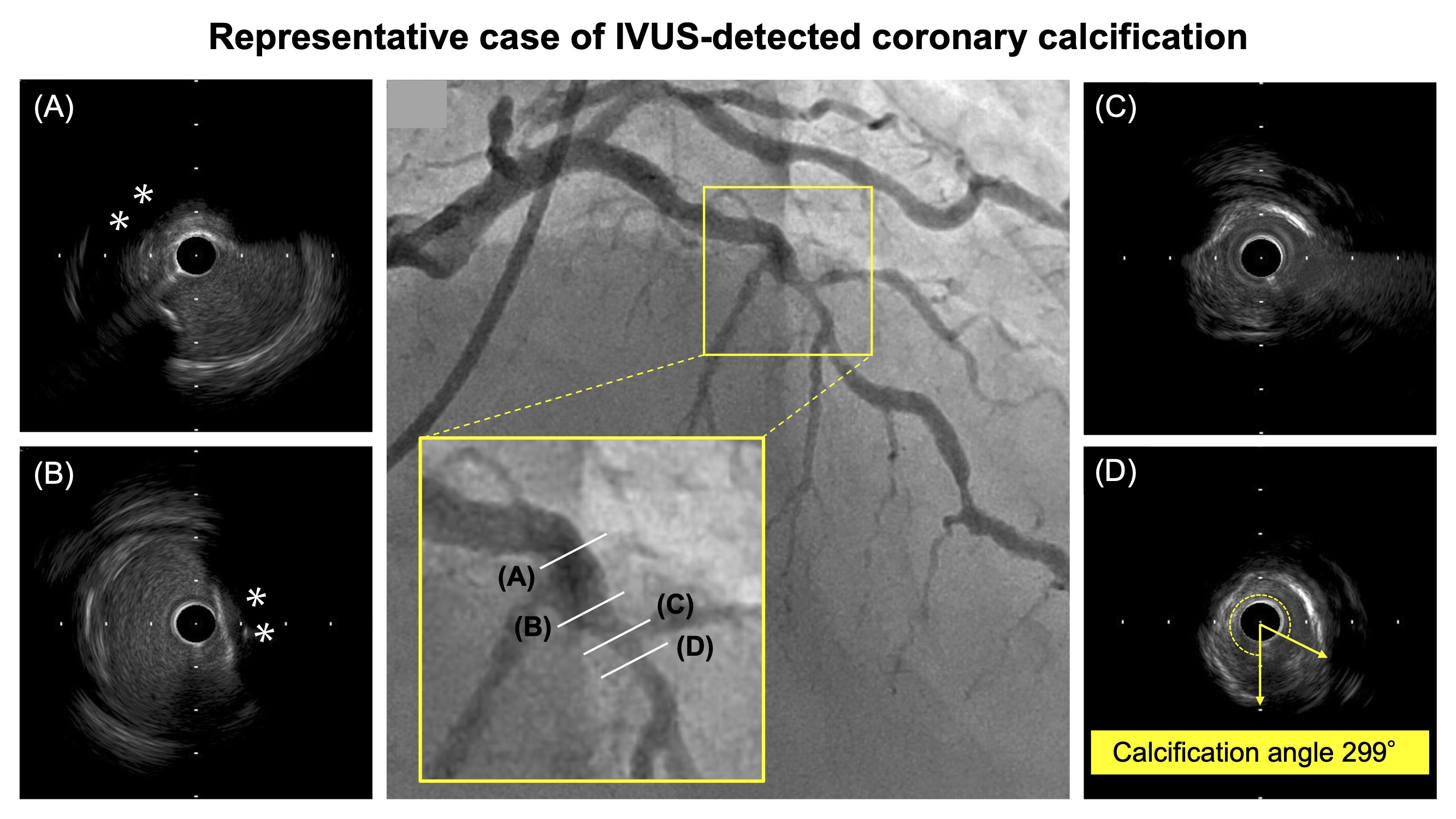

Supplement: Supplementary Figure S2 [file Image2.jpeg]
